# Exploration of Multi-Source Lignocellulose-Degrading Microbial Resources and Bioaugmentation Strategies: Implications for Rumen Efficiency

**DOI:** 10.3390/ani15131920

**Published:** 2025-06-29

**Authors:** Xiaokang Lv, Zhanhong Qiao, Chao Chen, Jinling Hua, Chuanshe Zhou

**Affiliations:** 1 College of Animal Science, Anhui Science and Technology University, Fengyang 233100, China; lvxk@ahstu.edu.cn (X.L.); 18731310109@163.com (Z.Q.); chenchao@ahstu.edu.cn (C.C.); 2 Hunan Provincial Key Laboratory of Animal Nutritional Physiology and Metabolic Process, National Engineering Laboratory for Pollution Control and Waste Utilization in Livestock and Poultry Production, Institute of Subtropical Agriculture, Chinese Academy of Sciences, Changsha 410125, China

**Keywords:** lignocellulose degrading bacteria, rumen microorganisms, metagenomics, cellulase, gene editing technology

## Abstract

Utilizing straw feed is an effective strategy to optimize straw resource utilization by incorporating microbial degradation agents to expedite lignocellulose breakdown and enhance feed efficiency. Lignocellulose-degrading species and microbial communities are present in various Earth ecosystems, including the rumen of ruminants, insect digestive tracts, forest soil, and microbial populations in papermaking processes. The rumen of ruminants harbors a diverse range of microbial species, making it a promising source of lignocellulose-degrading microorganisms. Exploring alternative systems like insect intestines and forest soil is essential for future research. Current studies primarily rely on traditional microbial isolation techniques to identify lignocellulose-degrading strains, underscoring the necessity to transition to utilizing microbial culturomics and genome-editing technologies for discovering and manipulating cellulose-degrading microbes. This review provides an overview of lignocellulose-degrading microbial communities from diverse environments, encompassing bacterial and fungal populations. It also delves into the use of metagenomic, metatranscriptomic, and metaproteomic approaches to pinpoint highly efficient cellulase genes, along with the application of genome-editing tools for engineering lignocellulose-degrading microorganisms. The primary objective of this review is to offer insights for further exploration of potential lignocellulose-degrading microbial resources and high-performance cellulase genes to enhance roughage utilization in ruminant rumen ecosystems.

## Simple Summary

This review explores the diverse world of microbes (bacteria and fungi) capable of breaking down lignocellulose, found in environments from cow rumens to insect guts and forest soils. We cover how researchers identify these microbes and, crucially, the shift towards using advanced multi-omics techniques to pinpoint highly efficient straw-degrading enzymes, alongside genome-editing tools to engineer improved microbes. Our aim is to guide the discovery of new microbial resources and superior enzymes to enhance the utilization of roughage like straw, especially within ruminant feeding systems.

## 1. Introduction

The global availability of straw resources is substantial, with an annual production exceeding 7 billion tons worldwide, of which China contributes 25% [1]. Currently, crop straw is primarily utilized through various methods, including direct field incorporation (3–5%), composting for field application (7–9%), livestock feed (8–11%), organic fertilizer production (2–5%), edible fungi cultivation (4–5%), inefficient combustion (approximately 15%), among others [2]. Although straw combustion is widespread, it results in significant resource loss and environmental harm [3]. Given the growing global demand for cattle and sheep feed, converting straw into animal feed offers a sustainable and productive alternative [4]. Lignocellulose, a ubiquitous substance on Earth, is the primary component of straw, constituting a significant portion of plant cell walls in various plant tissues. The lignocellulose content in straw varies with plant species. Wheat straw contains 32–45% cellulose, 20–45% hemicellulose, and 11–26% lignin [5]. Corn stalk contains cellulose 36.89%, hemicellulose 20.42%, and lignin 17.38% [6]. Cellulose and hemicellulose of straw are 50–80% of dry matter, and lignin is 10–15% of dry matter [7]. The complex structure of lignocellulose makes it inherently resistant to natural degradation. Consequently, there is a continuous research focus on improving the efficient breakdown of lignocellulose. Microorganisms capable of cellulose degradation produce cellulases, which facilitate the conversion of lignocellulose into mono- or oligosaccharides, thereby enhancing the nutritional value of straw for animal feed [8].

The recent rapid progress in biotechnology has sparked interest in identifying effective lignocellulose-degrading microorganisms for straw feed processing [9,10,11]. Significant headway has been achieved in isolating and characterizing highly efficient lignocellulose-degrading microorganisms [12]. Techniques such as metagenomics and microbial culturomics have successfully identified potent cellulose-degrading microorganisms from various environments, including insect guts [13], ruminant gastrointestinal tracts [14], and forest soils [15]. Moreover, the integration of metagenomic [9], metatranscriptomic [10], and metaproteomic methodologies [11] has substantially broadened the pool of cellulase genes. The isolation of these potent cellulose-degrading microorganisms and the elucidation of cellulase genes serve as valuable microbial assets for straw feed processing. The genetic manipulation of cellulose-degrading microorganisms is expected to be a crucial technical approach for enhancing cellulose utilization in the future. This review provides a comprehensive summary of recent progress in isolating cellulose-degrading microorganisms, identifying cellulase genes, and genetically modifying cellulose-degrading strains. Utilizing multi-omics technology and gene editing is essential for enhancing the cellulose degradation capacity of cellulose-degrading bacteria. This review broadens the scope of cellulose-degrading bacterial sources, emphasizing the investigation of the extreme environmental adaptability of these bacteria as a crucial area for future research. Such efforts have the potential to significantly enhance the global utilization of agricultural waste and straw feed.

## 2. Lignocellulose Degradation Mechanisms

Lignocellulose is primarily composed of cellulose (45–50%), hemicellulose (20–30%), and lignin (20–30%) [16].

### 2.1. Degradation Mechanism of Lignin

Lignin is a complex and heterogeneous polyphenolic polymer. Enzymes responsible for lignin degradation primarily include lignin peroxidase (LiP), manganese peroxidase (MnP), and laccase [17]. LiP, containing a heme group, aids in substrate oxidation by utilizing hydrogen peroxide or similar peroxides as co-substrates. This peroxidase catalysis leads to the electron loss of aromatic rings, resulting in the formation of aryl cation radicals that subsequently degrade into smaller molecules in the presence of molecular oxygen [18]. MnP, an oxidoreductase containing heme and Mn^2+^ in its catalytic center, initiates its catalytic cycle through the oxidation of Mn^2+^ to Mn^3+^ by hydrogen peroxide during the reaction. The Mn^3+^ chelate acts as an oxidant, directly targeting phenolic structures within lignin. Laccase, a copper-containing polyphenol oxidase, utilizes molecular oxygen as an electron acceptor to oxidize both phenolic and non-phenolic compounds, producing free radicals while converting oxygen to water. With a wide substrate range, laccase catalyzes the oxidation of various compounds such as phenols, aromatic amines, and aliphatic amines [19]. These enzymes exhibit substrate specificity: laccase and MnP preferentially degrade phenolic structures, whereas LiP targets non-phenolic lignin components.

### 2.2. Degradation Mechanism of Cellulose

Cellulose, a linear macromolecular polymer comprising β-1,4-glycosidic linkages of D-glucose units, is insoluble in water and common organic solvents [20]. It is the predominant component of plant cell walls, where cellulose chains are connected by hydrogen bonds [21]. Cellulose accounts for over 50% of the carbon content in plants, making it the most abundant organic material in nature. Its strong structural integrity and stability make it highly resistant to digestion and absorption by most organisms [22].

Cellulases are predominantly synthesized by microorganisms, encompassing bacteria, fungi, and protozoa. These enzymes operate within a system comprising three key elements: endo-β-1,4-glucanase (EG), cellobiohydrolase (CBH), and β-glucosidase (BGL). Endoglucanases catalyze the breakdown of β-1,4-glycosidic bonds present in cellulose chains, disrupting the elongated chain structure and yielding free chain ends. Exoglucanases, exemplified by CBH, target these liberated chain ends, cleaving glycosidic bonds to release cellobiose units. Subsequently, β-Glucosidases further degrade cellobiose and short-chain oligosaccharides into glucose monomers. Extensive investigation has underscored the indispensable nature of the cooperative action of these three enzymes for thorough cellulose degradation [23]. Cellulose, hemicellulose, and other non-starch polysaccharides (NSPs) constitute principal anti-nutritional components in plant-based feed. Glycoside hydrolases, including cellulases, glucanases, xylanases, and mannanases, possess the capability to cleave the glycosidic bonds within NSPs, thereby mitigating their anti-nutritional properties and yielding low-molecular-weight products such as oligosaccharides or monosaccharides [24].

### 2.3. Degradation Mechanism of Hemicellulose

Hemicellulose undergoes biodegradation into monosaccharides and acetic acid. Xylan, a significant constituent of hemicellulose, displays intricate structural diversity, necessitating a complex enzymatic system for its breakdown. Efficient degradation of xylan-based hemicellulose requires the coordinated action of multiple enzymes. The enzymatic system responsible for hemicellulose degradation includes endo-β-1,4-xylanase, β-xylosidase, and α-L-arabinofuranosidase, collectively targeting most naturally occurring xylan-type hemicelluloses [25]. Specifically, endo-β-1,4-xylanase cleaves β-1,4-glycosidic bonds in the xylan backbone, producing xylooligosaccharides. Subsequently, β-xylosidase acts on the non-reducing ends of these oligosaccharides to liberate xylose residues [22].

## 3. Sources and Systems of Cellulose-Degrading Microorganisms

### 3.1. Insect Gut

**Figure 1:**
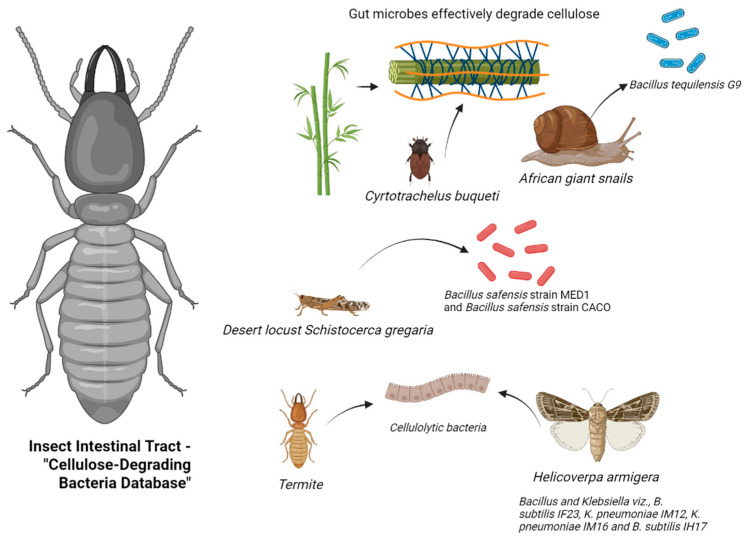
Database of insect gut cellulose-degrading bacteria (generated by bioRender).

The insect gut acts as a natural bioreactor, containing a rich reservoir of microbial communities for cellulose degradation. Various studies have highlighted the impressive lignocellulose-degrading capacities of different insect gut systems (Figure 1). For instance, Dar et al. [26] isolated *Bacillus tequilensis G9*, a cellulase-producing strain with high yield, from the hemolymph of African giant snails (Table 1). By combining 16S rRNA sequencing with enzyme activity assays (DNS method), the strain exhibited cellulase activity of 956.9 IU/mL on day 8 in a sugarcane bagasse-induced medium. This discovery not only expands the range of cellulolytic microorganisms but also provides new insights into identifying high-activity strains. Luo et al. [13] conducted a comprehensive assessment of bamboo weevils (*Cyrtotrachelus buqueti*) in degrading bamboo lignocellulose through both in vivo and in vitro experiments. The results demonstrated in vivo degradation efficiencies of 61.82%, 87.65%, and 69.05% for cellulose, hemicellulose, and lignin, respectively. In contrast, in vitro efficiencies were lower at 24.98%, 37.52%, and 26.67%. This significant difference emphasizes the crucial regulatory function of the insect gut microenvironment in cellulose degradation mechanisms.

Nelson et al. isolated two highly efficient cellulose-degrading bacterial strains from the gut of desert locusts. The cellulolytic activity index (ICA) of *Bacillus safensis MED1* and *Bacillus* sp. *CACO* was determined as 1.146 ± 0.109 and 0.8442 ± 0.09203, respectively, using the agar plate clearance zone method. Oktiarni et al. [27] screened 24 cellulose-degrading strains from the gut of peatland termites (*Macrotermes* sp.), with strains 37 (*Staphylococcus* sp.), 39 (*Microbacterium* sp.), 40 (*Bacillus* sp.), and 62 (*Brevibacterium* sp.) exhibiting cellulolytic indices of 3, 4, 2.5, and 2.5, respectively. Biochemical characterization confirmed these strains as catalase-positive and citrate-positive but negative for indole and urease tests. Dar et al. [28] demonstrated that the foregut of cotton bollworms (Helicoverpa armigera) is the primary enrichment site for cellulose-degrading microorganisms. Isolation and cultivation yielded 71 bacterial strains, with 54% isolated from the foregut. Sodium carboxymethyl cellulose (CMC-Na) plate assays revealed significant cellulose-degrading activity in 25 strains belonging to the phyla *Firmicutes* and *Proteobacteria*.

**Table 1:** Isolation of cellulose-degrading bacteria from the intestinal tract of insects.

Year	Host Source	Cellulose-Degrading Bacteria	Microbe Phylum, Genus	References
2018	*Achatina fulica*	*B. tequilensis G9*	*Firmicutes*	[26]
2019	*Bamboo weevils*	No specific strain was identified	—	[13]
2021	*Desert Locust*	*Bacillus safensis MED1*, *Bacillus* sp. *CACO*	*Firmicutes*	[29]
2021	*Indralaya*	*Staphylococcus* sp. *Microbacterium* sp. *Bacillus* sp. *Brevibacterium* sp.	*Staphylococcus*, *Microorganisms*, *Bacillus*, *Brevibacterium*	[27]
2021	*Cotton bollworm*	25 strains (*Firmicutes*, *Proteobacteria*)	*Firmicutes and Proteobacteria*	[28]

Bacterial strains exhibiting cellulolytic activity, identified from studies on bamboo weevils (*Cyrtotrachelus buqueti*), desert locusts, peatland termites (*Macrotermes* sp.), Giant African land snail, and cotton bollworms (*Helicoverpa armigera*).

### 3.2. Ruminant Gastrointestinal Tract

**Figure 2:**
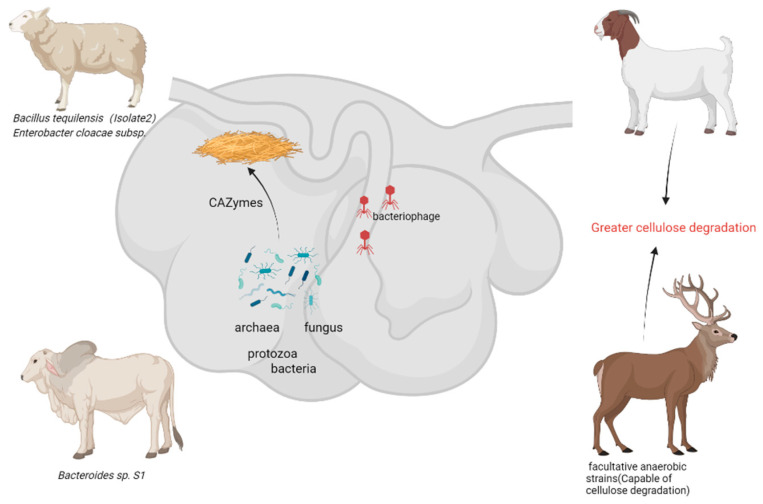
Cellulose-degrading bacteria from rumen of ruminants—potential resources for the future (generated by bioRender).

Ruminants predominantly consume cellulose-rich forage, with their rumen serving as a natural anaerobic fermentation system housing abundant cellulose-degrading microbial populations. Research indicates that the rumen microbiota demonstrates a substantial biomass, consisting of bacteria (10^10^–10^11^ cells/mL), fungi (10^3^–10^6^ cells/mL), protozoa (10^4^–10^7^ cells/mL), phages (10^7^–10^9^ particles/mL), and archaea (10^6^ cells/mL) [30]. These microorganisms collaboratively break down cellulose into glucose for the host’s benefit. Notably, the *phyla Bacteroidetes* and *Firmicutes* are prevalent in the core rumen microbiota, collectively representing over 80% [31]. The rumen microbial genome encodes a wide array of carbohydrate-active enzymes (CAZymes), establishing it as a crucial repository for novel cellulases (Figure 2).

#### 3.2.1. Cellulose-Degrading Microorganisms from Cattle Rumen

Sari et al. [14] isolated a highly cellulolytic strain, *S1*, from the rumen fluid of Aceh cattle in Indonesia. This strain demonstrated notable cellulolytic activity on BHM-CMC agar medium (cellulolytic index > 1, clearance zone diameter 2.5 cm). Identified as a Gram-negative bacillus, it exhibited optimal growth conditions at pH 6.0–6.5 and 36–39 °C (Table 2). Pang et al. [32] isolated a cellulolytic strain, *ZH4*, from the rumen of Inner Mongolia cattle. Through morphological, physiological, and biochemical characterization, as well as 16S rDNA gene sequencing, the strain was identified as *Escherichia coli*. Analysis of extracellular enzyme activity revealed that the strain exhibited cellulase production. Specifically, at pH 6.8, the exoglucanase activity was measured at 9.13 IU, endoglucanase activity at 5.31 IU, and β-glucosidase activity at 7.27 IU. Furthermore, when utilizing corn stalk as a substrate, the strain produced 0.36 g/L ethanol and 4.71 mL/g hydrogen. The degradation rates of cellulose and hemicellulose were determined to be 14.30% and 11.39%, respectively [32].

Zhao et al. [33] utilized metagenomics to demonstrate that the yak rumen microbiota not only significantly increases the relative abundance of cellulolytic bacteria but also enhances the expression of cellulase and xylanase-encoding genes. Furthermore, the rumen microbial community enriches *succinate-metabolizing bacteria*, facilitating pyruvate fermentation to propionate and enhancing energy utilization efficiency.

#### 3.2.2. Cellulose-Degrading Microorganisms from Sheep Rumen

Guder et al. [34] evaluated 20 cellulose-degrading strains isolated from the sheep rumen, with 8 strains displaying significant cellulose degradation capability, notably Enterobacter cloacae subsp. *strain KLCD08* exhibiting the highest enzymatic activity. Ben Ghalib et al. [35] identified five highly cellulolytic strains from the sheep gastrointestinal tract using MALDI-TOF MS and 16S rRNA sequencing. These strains were classified under *Bacillus*, *Lysinibacillus*, and *Priestia genera*, with *Bacillus tequilensis (Isolate 2)* demonstrating the most pronounced cellulase activity.

Yang et al. [36] investigated rumen microorganisms in Wujimu sheep, identifying 22 isolates through 16S rDNA or ITS sequencing. Phylogenetic analysis revealed that these isolates were classified into four phyla: *Actinomycota, Firmicutes, Proteobacteria*, and *Ascomycota*. Notably, *Bacillus subtilis RE251* exhibited remarkable enzyme activities (including endoglucanase, exoglucanase, and β-glucosidase) during the degradation of alfalfa hay. After 10 days, this strain significantly disrupted the fiber structure, demonstrating promising practical application.

#### 3.2.3. Cellulose-Degrading Microorganisms from Other Ruminants

Tulsani et al. [37] successfully isolated *Aspergillus sydowii C6d* from the rumen of camels. Their analysis of CAZymes demonstrated that *C6d* expressed a total of 543 polysaccharide-degrading CAZymes, with 148 of these enzymes potentially participating in lignocellulose breakdown. Comparative genomic analysis between *C6d* and 30 widely studied lignocellulolytic fungi, including *white rot fungi*, *brown rot fungi*, and *soft rot fungi*, revealed a notable enrichment of cellulolytic and pectinolytic CAZymes in the *C6d* genome when compared to other fungal species.

Pramartaa et al. [38] identified five facultative anaerobic strains capable of degrading cellulose from the feces of Southeast Asian banteng, Javan banteng, muntjac, and Timor deer. The cellulolytic indices indicated high cellulolytic activity (index ≈ 1.2) in isolates from Southeast Asian and Javan banteng feces, suggesting their promise as cellulose-degrading candidates.

**Table 2:** Isolation of cellulose-degrading bacteria from rumen in ruminants.

Year	Host Source	Isolated Cellulose-Degrading Bacteria	Microbes Phylum, Genus	References
2017	Indonesian Aceh bovine rumen	*Bacteroides* sp. *S1*	Probably a new species of Enterobacteriaceae.	[14]
2017	Cattle rumen	*ZH-4*	*Proteobacteria*	[32]
2022	Rumen microbial community in yak	Uncultured microbiota	—	[33]
2019	Sheep rumen	*Enterobacter cloacae* subsp. *KLCD08*	*Genus Bacillus*	[34]
2024	Sheep digestive tract	*Bacillus tequilensis Isolate2*	*Bacillus*, *Lysinibacillus*, *Priestia*	[35]
2022	Wujimu Sheep	*22 isolates*	*Actinomycetales*, *Firmicutes*, *Proteobacteria* and *Ascomycetes*	[36]
2022	Camel rumen	*Aspergillus sydowii C6d*	*Ascomycota*	[37]
2024	The feces of Southeast Asian banteng, Javan banteng, muntjac, and Timor deer.	*Five facultative anaerobic strains*	—	[38]

Representative bacteria isolated from the rumen of diverse ruminants (cattle, sheep, yak, camel, deer, banteng) demonstrating significant cellulolytic potential. Highlighted strains exhibit high cellulolytic indices, enzymatic activities (endoglucanase, exoglucanase, β-glucosidase), and lignocellulose degradation capabilities, positioning the rumen microbiome as a key resource for future biotechnological applications (especially for ruminant animals such as goats, yaks, and deer that are tolerant to roughage).

### 3.3. Forest Soil-Derived Cellulose-Degrading Microorganisms

Forest soil is a crucial habitat for cellulose-degrading microorganisms due to the abundance of cellulose substrates in the thick layer of decaying plant litter. Several research groups have isolated and characterized highly efficient cellulose-degrading strains from diverse forest soils. For instance, Mokale Kognou et al. [39] utilized a carboxymethyl cellulose (CMC)-Congo red screening method to isolate six cellulose-degrading strains from mixed soil samples collected on a Canadian university campus (Table 3). Through 16S rRNA sequencing, *Bacillus* sp. *MKAL6* exhibited the highest cellulase activity. Optimization studies using Box–Behnken design revealed that the addition of 10 mM sucrose significantly enhanced cellulase activity. SDS-PAGE analysis indicated that the cellulases produced by this strain had molecular weights concentrated around 25 kDa, suggesting a potential link to the enzyme’s efficient catalytic structure. In a separate study, Ma et al. [40] isolated 55 cellulolytic bacterial strains from decaying wood in the Qinling Mountains, with *Methylobacillus* sp. 1EJ7 demonstrating optimal degradation efficiency for wheat straw, corn stover, and switchgrass. Naresh et al. [41] isolated seven thermophilic cellulose-degrading strains from Malaysian tropical forest soil, with *Anoxybacillus* sp. *UniMAP-KB06* exhibiting the highest hydrolytic activity. Additionally, Khosravi et al. [42] identified *Brevibacillus borstelensis strains US5* and *US7* from forest soil in Kerman, Iran, which maintained high cellulase activity even at 65 °C, indicating their potential for industrial applications.

Du et al. [43] isolated *Massilia* sp. *NEAU-DD11T*, a novel cellulose-degrading strain, from rice rhizosphere soil using polyphasic taxonomic approaches. This Gram-negative bacillus was characterized through various methods, including 16S rRNA gene sequence analysis (>98.4% similarity to Massilia species), whole-genome phylogeny, fatty acid profiling (predominantly hexadecanoic acid (C16:), cycloheptadecanoic acid (C17:-cyclo), and hexadecenoic acid (C16:1ω7c)), and polar lipid composition (e.g., diphosphatidylglycerol, phosphatidylglycerol). Ma et al. [15] identified seven potential cellulolytic strains from soil using 16S rRNA sequencing. Through DNS-based enzyme activity screening, two highly efficient strains, *Rhodococcus wratislaviensis YZ02* and *Pseudomonas anthosomatis YZ03*, were selected, with genome sizes of 8.51 Mb (8466 genes) and 6.66 Mb (5745 genes), respectively. Analysis of CAZyme revealed the presence of glycoside hydrolase (GH) family genes and other cellulose degradation-related genes in both strains.

Lam et al. [44] isolated *Robertkochia solimangrovi* from mangrove soil and conducted genomic analysis, revealing 87 lignocellulose-degrading enzymes. These enzymes include cellulases (GH3, GH5, GH9, and GH30), xylanases (GH5, GH10, GH43, GH51, GH67, and GH115), mannanases (GH2, GH26, GH27, and GH113), and xyloglucanases (GH2, GH5, GH16, GH29, GH31, and GH95). *R. solimangrovi* exhibits the capability to produce lignocellulolytic enzymes for the decomposition of empty fruit bunches (EFB) from oil palm. The integration of genomic analysis with experimental investigations has elucidated the potential of *R. solimangrovi* as a valuable candidate for the seawater biorefinery sector.

Hao et al. [45] isolated cellulose-degrading strains from humus soil in cold regions through a screening process involving sodium carboxymethyl cellulose culture and Congo red staining. Further characterization of cellulase activity included morphological observation, physiological and biochemical tests, and phylogenetic analysis of internal transcribed spacer sequences. A cold-active cellulose-degrading strain (LC-6) belonging to Tausonia pullulans was identified, exhibiting peak activities of 4.93 U/mL for carboxymethyl cellulase, 7.62 U/mL for cellobiohydrolase, 14.17 U/mL for β-glucosidase, and 7.15 U/mL for filter paper enzyme at temperatures ranging from 10 °C to 15 °C. Following fermentation at 15 °C for 15 days, LC-6 achieved degradation rates of 25.01% for hemicellulose, 16.13% for cellulose, and 24.35% for lignin. These findings demonstrate the efficient degradation of straw by strain LC-6 at low temperatures, highlighting its significant potential in lignocellulosic biomass utilization.

### 3.4. Other Source Systems

**Figure 3:**
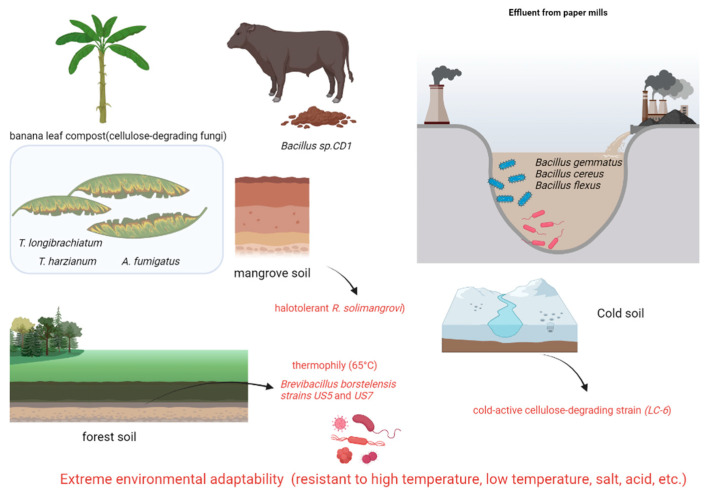
Cellulose-degrading bacteria from different sources (extreme environmental adaptability) (generated by bioRender).

Apart from traditional sources, specialized ecological niches harbor microorganisms with highly effective cellulose-degrading capabilities. These strains hold significant promise for applications in treating agricultural waste, purifying industrial wastewater, and converting organic waste (Table 4). Notably, cellulolytic microorganisms naturally decompose cellulose-rich waste tissues (e.g., leaves, pseudostems) from banana plantations [46]. Legodi et al. [47] identified six highly efficient fungal strains, such as *Trichoderma longibrachiatum*, *T. harzianum*, and *Aspergillus fumigatus*, from compost. These strains displayed significant endoglucanase activity on carboxymethyl cellulose (CMC)-Congo red agar plates, with *T. harzianum* exhibiting the highest total cellulase activity at 28.6 U/mL. Sinza et al. [48] identified five bacterial strains with complete cellulase systems out of 17 isolates from paper mill wastewater, including *Bacillus sporothermodurans*, *B. cereus*, and *B. flexus*. Among these, *B. flexus* showed the highest cellobiose degradation efficiency (89% degradation rate at 72 h) with optimal activity observed at pH 6.5–7.0. Demissie et al. [49] compared the distribution of cellulose-degrading microbes in various environments such as forest soil, cattle dung, brewery waste, and industrial refuse. *Bacillus* sp. *CD1* isolated from cattle dung exhibited the highest enzymatic activity, generating a 4.5 mm clearance zone and a cellulolytic index of 3.1. This strain demonstrated peak activity under conditions of 1% CMC, 40 °C, and pH 7, highlighting livestock feces as a rich source for isolation. In contrast, only a limited number of active strains were isolated from brewery waste and industrial refuse, with no efficient degraders found in forest soil samples. These investigations broaden the possibilities for acquiring cellulose-degrading microorganisms: strains derived from agricultural waste are conducive to bio-composting, those from industrial sources demonstrate robust environmental resilience for wastewater treatment, and strains isolated from livestock feces possess comprehensive enzyme systems suitable for industrial-scale cultivation. Subsequent research should emphasize the exceptional environmental adaptability of these distinct strains, the synergistic mechanisms of multi-enzyme systems, and the enhancement of large-scale cultivation techniques (Figure 3).

Representative bacteria and fungi sourced from non-traditional environments, including forest soil, decaying wood, cold humus, mangrove soil, compost, industrial wastewater (paper mill), livestock dung, and agricultural waste. Key isolates exhibit exceptional tolerance to extremes: thermophily (65 °C), psychrophily (active ≤ 15 °C), halotolerance (mangrove), and varied pH. This inherent adaptability, coupled with significant lignocellulose degradation capabilities (e.g., cold-active *LC-6*, thermostable *Brevibacillus borstelensis US5/US7*, halotolerant *R. solimangrovi*), positions these strains as valuable resources for applications in bio-composting, wastewater treatment, biomass conversion, and biorefineries under challenging conditions.

## 4. Mining and Separation Techniques for Cellulose-Degrading Bacteria

### 4.1. Screening Strategies for Aerobic and Anaerobic Bacteria

Isolation techniques are customized based on the physiological characteristics of the target microbial strains. The streak plate method is commonly utilized for aerobic bacteria [50]. This involves enrichment culture, serial dilution, and streaking on selective media (e.g., cellulose-specific media like CMC-Na or filter paper-based media) until pure colonies are obtained. Anaerobic strains necessitate oxygen-free environments [51]. Lanjekar et al. [52] demonstrated a standard anaerobic isolation procedure using rumen samples from Pune cattle. Enrichment in PYGR broth under anaerobic conditions, followed by 16S rRNA sequencing (GenBank alignment via MAFFT) and phylogenetic tree analysis, identified Actinomyces sp. CtC72T as an obligate anaerobe. *Strain CtC72T* displayed a generation time of 2.7 h in PYGR broth under optimal conditions (48 h incubation, no aerobic growth observed).

### 4.2. Microbial Culturomics

The use of multiple culture conditions combined with high-throughput sequencing technology to identify the isolated strains is defined as culturomics. Compared to traditional microbial isolation and culture methods, the high-throughput and parallel nature of culturomics has earned it the status of “omics”. Previously, traditional microbial culture methods were constrained by the complex nutritional requirements and interactions of microorganisms, resulting in more than 99% microorganisms in environmental samples remaining uncultured [53]. Lagier et al. [54] introduced culturomics into the field of environmental microbiology, enabling the visualization and functional analysis of uncultivable microbial resources. Currently, the primary technologies employed in culturomics fall into the following two categories: (1) droplet-based microfluidics; (2) microarray-based technology. Droplet-based microfluidics leverage two-phase fluid systems to encapsulate individual microbial cells in biocompatible oil droplets, enabling high-throughput screening and isolation under controlled conditions. Due to its advantages in high throughput, parallelization, standardization, and dynamic process, this technology is considered a key component in the workflow of single-cell microbial isolation and culture [55]. However, microorganisms that thrive in anaerobic environments, such as the rumen and intestines, encounter additional challenges when using microfluidics for the separation, culture, and analysis of individual microbial cells. Specifically, sample processing, growth detection, and microfluidic sorting must be conducted in anaerobic conditions [56]. In contrast to microfluidic-based culturomics, microarray-based technology offers a larger microbial culture space, enabling the expansion of single cells into larger volumes of high-density microbial colonies. Compared to microfluidics, separation of selected microcolonies from discrete microcompartments is generally more straightforward. At present, culturomics is infrequently employed in rumen microbial research; however, existing studies have already demonstrated its enormous potential in reducing the unknown range of rumen microbial species and exploring rumen microbial functions. Zehavi et al. [57] identified that various factors, including culture medium type, sample dilution, and phylogenetic relationships, can affect the cultivability of rumen microorganisms, with sample dilution notably increases the possibility of isolating ‘uncultured’ microorganisms. Additionally, microbial abundance and phylogenetic relationships are the primary determinants of the cultivability of rumen microorganisms. Rumen microorganisms are intricately associated with essential functions such as polysaccharide degradation, protein hydrolysis and synthesis, cellulose degradation, and rumen detoxification. Consequently, rumen microbial purification and cultivation based on culturomics can isolate certain key functional strains, thereby providing new insights into the rumen microbiome [53].

Culturomics not only expands microbial genetic resources but also modifies the composition and abundance of microbial communities in specific environments. The physiological health of hosts and the occurrence of diseases are closely related to changes in the composition of the gut microbiota. By transplanting strains isolated through culturomics, the structure of microbiota can be regulated, thereby playing a crucial role in restoring microbial homeostasis, with the aim of treating clinical diseases of the digestive tract in ruminant animals. Given the potential of microbial culturomics in restoring microbial balance and correcting microbial composition, isolating and culturing key microorganisms associated with diseases may provide insights into microbial communication mechanisms under specific environmental conditions.

Specialized techniques are essential for isolating cellulose-degrading bacteria. The emerging field of culturomics, which merges high-throughput culture and sequencing methods, has revolutionized microbial isolation by facilitating the growth of previously deemed “unculturable” species. Technological advancements such as microfluidics and microarray-based culturomics have bolstered efficiency, although challenges persist in anaerobic cultivation. This strategy is particularly advantageous for investigating the rumen microbiome, discovering novel cellulose-degrading strains, and elucidating their roles in biomass degradation. Future integration with multi-omics approaches will deepen understanding of microbial functions, propelling progress in biofuel production, waste management, and animal nutrition. Continuous advancements in microbial isolation are pivotal for unlocking further biotechnological and medical prospects.

## 5. Classification of Major Cellulose-Degrading Microorganisms

### 5.1. Bacteria

#### 5.1.1. *Fibrobacter succinogenes*

Succinic acid, also known as butanedioic acid, is a crucial industrial chemical synthesized biologically through pathways involving *Fibrobacter succinogenes*, a strictly anaerobic Gram-negative bacterium originally isolated from the bovine rumen. This bacterium possesses unique capabilities in breaking down complex cellulose substrates such as straw [58]. *F. succinogenes* metabolizes cellulose, cellobiose, and glucose to produce acetate, succinate, and formate via anaerobic fermentation. Of particular note is its succinic acid biosynthesis pathway, which holds considerable industrial significance. Research has shown that *F. succinogenes*, a predominant cellulolytic species in the rumen, effectively degrades cellulose and hemicellulose in straw by secreting a combination of hydrolytic enzymes.

Metagenomic analyses have unveiled that the genome of *F. succinogenes* not only contains cellulase systems but also essential enzymes like pectinases, glucanases, arabinogalactanases, and xylanases. This array of enzymes works synergistically to efficiently break down intricate plant cell wall structures, particularly the interconnected network of cellulose, hemicellulose, and pectin.

P.C. Lee et al. [59] isolated a new bacterium capable of producing succinic acid from the rumen of cattle. This bacterium, named *Mannheimia succiniciproducens MBEL55E*, is a non-motile, non-spore-forming, mesophilic, and capnophilic Gram-negative bacillus or coccobacillus. Through phylogenetic classification based on 16S rRNA sequencing and physiological assessments, it was assigned to the redefined *genus Mannheimia*. Thriving within a pH range of 6–7.5 under conditions of less than 100% CO_2_, this bacterium consistently produces succinic acid, acetate, and formate in a fixed ratio of 2:1:1. When cultivated anaerobically with 20 g/L glucose as the carbon source, this strain yielded 13.5 g/L of succinic acid.

Cellulase preparations have proven effective as feed additives, enhancing nutrient availability, fiber digestibility, and animal growth performance. Particularly, the recombinant expression of cellobiohydrolase genes sourced from *F. succinogenes* in lactic acid bacteria notably enhanced neutral detergent fiber degradation rates in corn stover silage.

#### 5.1.2. *Ruminococcus albus*

*Ruminococcus albus*, a predominant Gram-positive strict anaerobe in the rumen, relies on exogenous vitamins and growth factors for growth and demonstrates significant enzymatic activity in lignocellulose breakdown (Table 5).

In co-culture experiments, Yeoman et al. [60] observed spatially distinct microcolonies of *R. albus*, *Fibrobacter succinogenes*, and *Ruminococcus flavefaciens* on cellulose substrates. Introduction of *R. flavefaciens* led to a 43.2% decrease in *F. succinogenes*, yet overall cellulose degradation remained unaffected. Metatranscriptomic analysis revealed that *R. albus* predominantly expresses GH5 family enzymes (62.3% of total CAZymes), while *R. flavefaciens* relies on GH9 family enzymes (51.8%), suggesting functional complementarity through distinct enzymatic approaches to maintain efficient ruminal fiber degradation.

#### 5.1.3. *Ruminococcus flavefaciens*

*Ruminococcus flavefaciens*, a key cellulolytic bacterium in the rumen, plays a crucial ecological role in ruminant digestion by producing a diverse array of enzymes that effectively degrade cellulose, hemicellulose, and pectin. Recent research has shed light on its distinctive degradation mechanisms and potential applications [61]. Boonsaen [62] and colleagues isolated six *R. flavefaciens* strains from the rumen of swamp buffaloes, among which strain *OS14* exhibited the highest fiber digestibility, either in isolation or in co-culture with non-cellulolytic bacteria. Co-culturing *OS14* with strain *S137* resulted in optimal fiber degradation and a significant increase in acetate and propionate production.

Odenyo AA et al. [63] investigated the degradation of wheat straw (WS) and alkaline hydrogen peroxide (AHP)-treated wheat straw (AHPWS) by *Ruminococcus albicans No. 8* and *Flavobacterium pyogenes FD-1* through monitoring bacterial growth (OD600) and determining dry matter disappearance (DMD) of the substrate. In the presence of phenylpropionic acid (PPA) and phenylacetic acid (PAA) in the established medium, the degradation of AHPWS (39.6 ± 2.6%) was enhanced compared to AHPWS without PPA and PAA (24.9 ± 7.6%). However, there was no synergistic effect on degradation when both species were co-cultured with WS or AHPWS substrates. Specifically, PPA and PAA did not affect the disappearance of AHPWS by *Flavobacterium pyogenes FD-1*, nor was there any observed impact when both bacteria were cultivated together. Dry matter disappearance analysis in a complex medium revealed that *Aspergillus flavus FD-1* degraded AHPWS more rapidly (6.1 mg/d) than *Aspergillus kawachii 8* (4.2 mg/d).

### 5.2. Fungi

#### 5.2.1. White-Rot Fungi

*White-rot fungi* are recognized for their significant potential in selectively delignifying biomass due to their distinctive decomposing capabilities. Among *filamentous fungi*, *white-rot fungi* stand out as highly researched organisms, exhibiting exceptional efficacy and specificity in degrading lignocellulose. This unique feature substantially enhances the efficiency of lignocellulose degradation in the rumen [64].

*White-rot fungi* degrade lignin in roughage by employing enzymes such as lignin peroxidase, manganese peroxidase, multifunctional peroxidase, laccase, and hydrogen peroxide-generating enzyme. Manganese peroxidase and laccase specifically act on phenolic components of lignin [65]. In addition, cellulases, hemicellulases, and esterases are also thought to be extremely important in the degradation of lignocellulosic biomass [66] (Table 6).

Shrivastava et al. [67] conducted solid-state fermentation of wheat straw employing *white-rot fungi* to improve rumen digestibility and nutrient availability. After a 30-day fungal treatment, a significant reduction (*p* < 0.05) was noted in acid detergent fiber, neutral detergent fiber, hemicellulose, lignin, and cellulose components of the cell wall. This fungal fermentation of wheat straw resulted in a marked elevation in crude protein content, organic matter digestibility, short-chain fatty acid production, and metabolic energy, while concurrently lowering the C/N ratio.

Erven et al. [68] isolated intact lignin to investigate the vulnerability of structural motifs within wheat straw lignin to fungal degradation. Their findings indicate that the susceptibility of β-O-4′ aryl ethers to cleavage is influenced by the electronic density and local steric hindrance of the 4′-O-coupling ring. This sensitivity of β-O-4′ aryl ether substructures aligns with the lignin decomposition mechanism that is triggered by single electron transfer.

#### 5.2.2. Brown-Rot Fungi

*Brown-rot fungi*, obtained from decaying peach tissues, efficiently break down cellulose and hemicellulose while modifying lignin through demethylation, hydroxylation, and side-chain oxidation [69]. Genomic analyses have revealed a substantial simplification in the lignocellulose degradation system of *brown-rot fungi* during evolution, particularly in comparison to *white-rot fungi*, leading to a significant reduction in the abundance of cellulose-hydrolyzing and lignin-modifying enzymes [70].

*Brown-rot basidiomycetes* exhibit rapid cellulose depolymerization while preserving altered lignin residues. In a study by Jill Gaskell et al. [71], the analysis of Wolfiporia cocos transcriptomes and secretomes during growth on various substrates such as glucose, crystalline cellulose, aspen, or pine revealed significant gene upregulation on cellulose, aspen, and pine compared to glucose. Specifically, 30, 183, and 207 genes were upregulated by at least 4-fold on cellulose, aspen, and pine, respectively. Mass spectrometry analysis identified 64 glycoside hydrolase (GH) proteins, with 17 associated with upregulated transcripts on woody substrates, predominantly categorized as hemicellulases or chitinases. The high transcription levels of genes related to iron homeostasis, reduction, and extracellular peroxide generation support the cellulose depolymerization driven by reactive oxygen species (ROS), distinguishing it from the *brown-rot fungus* Postia placenta (Table 6).

Qi et al. [72] investigated the enzymatic decomposition and transformation of biomass by *white-rot fungi* (*Trametes versicolor*) and *brown-rot fungi* (*Gloeophyllum trabeum and Rhodonia placenta*). The study revealed that these fungi play a crucial role in breaking down wood components. Specifically, T. versicolor, a *white-rot fungus*, induces simultaneous decay and uniform biotransformation of wood constituents. In contrast, *brown-rot fungi* possess the capability to depolymerize carbohydrates and degrade polysaccharides via distinct biotransformation pathways, leading to diverse wood chemical transformations. Notably, both *white-rot* and *brown-rot fungi* pretreatments enhance wood cell porosity and accessibility, ultimately improving digestibility. Overall, the findings underscore the significant impact of these fungi on biomass degradation and transformation processes.

#### 5.2.3. Yeast

*Yeast* and its metabolites are effective functional feed additives that enhance livestock production performance and are commonly utilized in the industry. *Yeast* culture improves fiber degradation in the rumen of animals consuming cellulose-containing feed by utilizing free sugars to expedite the decomposition of fiber substances, thereby altering the fermentation process.

Comert et al. [73] investigated the impact of *yeast* supplementation on in situ dry matter degradability in lambs, as well as on rumen fermentation and growth performance. Their findings indicated that the group receiving *yeast* supplementation exhibited elevated rumen total volatile fatty acid (TVFA) levels. Moreover, the combination of ammonia treatment and *yeast* supplementation resulted in enhanced dry matter degradation efficiency, average daily weight gain, and NH^3^-N levels. The study concluded that the synergistic effect of ammonia treatment and *yeast* supplementation yielded superior enhancement in the feed value of wheat straw compared to either treatment in isolation. This combined approach was shown to enhance rumen fermentation efficiency and the potential productivity of ruminants.

Ben et al. [74] investigated the impact of Saccharomyces cerevisiae on the feed intake and nutrient digestibility of rams fed a wheat straw-based diet. Their findings revealed that, in comparison to the control group, the inclusion of Saccharomyces cerevisiae led to a significant increase in dry matter digestibility by 7.3% (*p* < 0.05), organic matter by 11.9%, and crude fiber by 24%. These results suggest that Saccharomyces cerevisiae has the potential to enhance nutrient digestibility in the diet of rams.

*Yeast* enhances fiber digestion and animal performance through various mechanisms, including the regulation of cellulose-degrading bacteria activity and rumen microbial populations. Research findings indicate that *yeast* additives facilitate early colonization of rumen microflora, leading to a notable increase in protozoa, *fungi*, and *lactic acid bacteria* populations [75]. Studies have identified yeast and its cultures as beneficial for improving fiber digestibility (Table 6).

## 6. Mining of Cellulose-Degrading Enzyme Genes

### 6.1. Metagenomics

Metagenomics, initially proposed by Handelsman in 1998, entails the extraction of total microbial DNA from environmental samples for subsequent sequencing. This method enables the direct identification of microbial functions, genomic relative abundance, and taxonomic resolution at the species level. By scrutinizing the genomes of pivotal cellulolytic strains, researchers can reconstruct potential functional pathways and modules to investigate microbial functionalities and genetic diversity. Metagenomics also unveils taxonomic profiles and relative abundances while delving into community-wide biological functions. Bai [76] utilized metagenomics to scrutinize the gut microbiota of Chinese bamboo rats (Rhizomys sinensis), known for their high-fiber diets. The investigation pinpointed abundant lignocellulose-degrading bacterial populations, with crucial functions associated with carbohydrate, amino acid, and nucleic acid metabolism. Carbohydrate esterases (CEs) and glycoside hydrolases (GHs) predominated among the CAZyme families in the cecum, primarily contributed by *Firmicutes*, *Bacteroidetes*, and *Proteobacteria*. A total of 587 CAZyme genes were identified, encompassing 7 CE and 21 GH families. Chai et al. [77] unearthed a novel bifunctional cellulase/hemicellulase (ZFYN184) from a soil metagenomic library. Sequence analysis categorized ZFYN184 into the GH44 family, featuring a single catalytic domain responsible for both endo-β-1,4-glucanase and β-mannanase activities. Meng et al. [9] characterized CelXyn2, a stable bifunctional cellulase-xylanase sourced from buffalo rumen metagenomes. CelXyn2 exhibited hydrolytic activity on rice straw, wheat straw, Leymus chinensis, and beet pulp, showcasing enhanced lignocellulose hydrolysis and in vitro rumen fermentation.

### 6.2. Metatranscriptomics

Although metagenome can identify potential functional genes, transcriptome can verify their actual expression. Macrotranscriptomics is a method used to extract all RNA from environmental samples collected at a specific time and location. This technique allows for the analysis of genome transcription and regulation at the transcriptional level, providing insights into the growth status and patterns of microorganisms, as well as enabling the investigation of the metabolic potential of microbial communities [78].

Fang et al. [10] investigated the utilization of corncob lignocellulose by Auricularia heimuer at various growth stages, analyzing the expression of CAZymes. They identified 46 differentially expressed CAZyme genes associated with lignocellulose degradation, demonstrating efficient cellulose utilization from corncobs. He et al. [79] studied the rumen microbiota of Hu sheep using metatranscriptomics, identifying *Firmicutes* and *Bacteroidetes* as the dominant phyla contributing to CAZyme expression. Among 14,489 genes annotated to 15 cellulase-containing GH families, GH3, GH5, and GH9 were the most prevalent. Two GH5 proteins (Cel5A-h28 and Cel5A-h11) showed high specific activities (222.2 and 142.8 U/mg) against carboxymethyl cellulose and p-nitrophenyl-β-d-cellobioside. Yu et al. [80] characterized fungal species in termite guts through internal transcribed spacer sequencing and genomic analysis. They identified *Talaromyces funiculosus*, an ascomycete containing an AA9 lytic polysaccharide monooxygenase (TfAA9A). Reverse transcription-PCR confirmed TfAA9A expression in termite guts, and heterologous expression in Pichia pastoris validated its cellulose-oxidizing capability.

### 6.3. Metaproteomics

**Figure 4:**
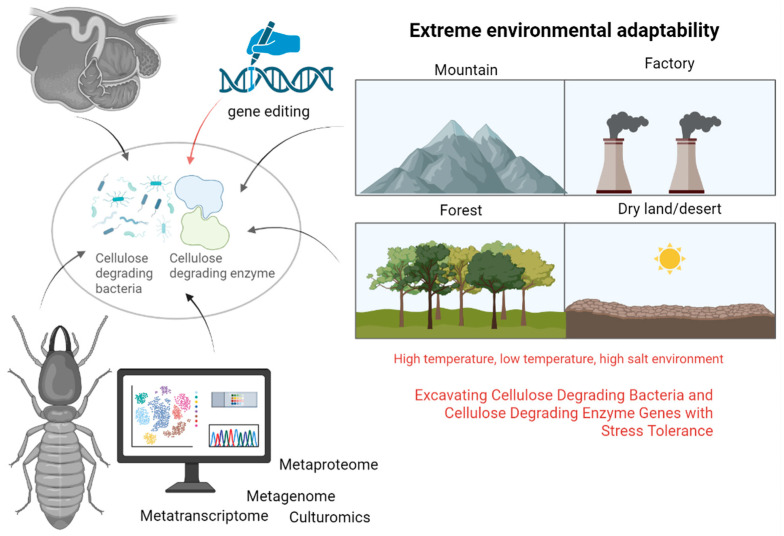
Multi-omics and gene editing methods for mining and modifying cellulose-degrading bacteria and enzymes (generated by bioRender).

Metaproteomics, the comprehensive protein profile of a microbial community in a specific environment at a particular time, represents an emerging field subsequent to metagenomics [81]. While genes and transcripts can predict functionality, protein abundance directly reflects functional activities within specific spatiotemporal contexts [82]. Wang et al. [11] conducted a study on bacterial and fungal diversity in mushroom residue compost using 16S rRNA sequencing and label-free quantitative proteomics. They identified 22,815 proteins, with cellulase-related proteins exhibiting stage-specific expression patterns that correlated with different composting phases. In a separate study, Toyoda et al. [83] employed metaproteomics to isolate cellulose-binding proteins (CBPs) from sheep rumen. Through SDS-PAGE and mass spectrometry analysis, they identified four CBPs from *Fibrobacter succinogenes*, which included a tetratricopeptide repeat domain protein, an OmpA family protein, a fibronectin domain protein, and cellulase F (EGF), all crucial for cellulose degradation in the rumen (Figure 4).

### 6.4. Gene Editing Technology

Recently, CRISPR/Cas9 systems have shown significant promise for genome manipulation in *filamentous fungi* and industrial microorganisms, particularly in coordinating the expression of multiple genes. Several investigations have successfully achieved the targeted evolution and effective production of enzymes that degrade lignocellulose using this method.

Liu et al. [84] have developed an efficient CRISPR/Cas9 system for multiplex genome engineering in *T. thermophila* and *M. heterothallica*. This system enables precise mutation of the amdS gene via NHEJ-mediated events. The cre-1, res-1, gh1-1, and alp-1 genes involved in cellulase production were targeted for editing. By a single transformation with the CRISPR/Cas9 system, up to four sites could be simultaneously disrupted through neomycin selection marker integration. Using this tool, multiple strains were created, showing significantly enhanced cellulase production, with extracellular secretory protein and lignocellulase activity increasing by up to 5-fold and 13-fold, respectively, compared to the wild-type strain.

Liu et al. [85] markedly improved the cellulose degradation capacity of *Bacillus subtilis RLI2019* by employing CRISPR/Cas9-mediated multi-gene integration. Specifically, eglS, cel48S, and bglS genes were successively integrated into the aprE, epr, and amyE loci of *Bacillus subtilis RLI2019*, resulting in the creation of the optimized strain *Bacillus subtilis AEA3*. This engineered strain demonstrates efficient degradation of crop straw, leading to the production of monosaccharides.

*Bacillus licheniformis 2709* underwent marker-free genome editing to disrupt the natural lchAC gene linked to foam production and the eps cluster responsible for extracellular mucopolysaccharides. Zhou et al. [86] optimized the expression of the alkaline protease gene (aprE) by evaluating various modular plasmids and genomic loci to identify the most efficient expression system. The study revealed that genomic expression of aprE outperformed plasmid expression. By optimizing the host and integrating the gene near the replication origin on the chromosome, the transcription level of aprE increased by 1.67-fold, leading to a significant 62.19% enhancement in enzyme activity compared to the wild-type strain producing alkaline protease.

## 7. Conclusions and Perspectives

Cellulose-degrading microorganisms, such as anaerobic bacteria in the rumen of ruminants, symbiotic bacteria in insect intestines, *white-rot fungi*, and *brown-rot fungi*, are prevalent in nature. Recent advancements in multi-omics technologies have facilitated a comprehensive analysis of the degradation mechanisms of these microbial celluloses. Techniques such as macrogenomics, macrotranscriptomics, and macroproteomics have been instrumental in elucidating these processes. Moreover, gene editing tools like CRISPR/Cas9 have been employed to enhance the cellulose-degrading capabilities of bacteria, leading to notable improvements in enzyme activity and substrate utilization efficiency. The utilization of complex microbial consortia in straw feed has yielded significant advancements in this field.

In the future, there is a need to focus on harnessing microbial resources from extreme environments. This includes identifying strains with high salt tolerance and low-temperature resilience, isolating cellulose-degrading bacteria from ruminants’ rumen, and addressing challenges related to anaerobic bacteria that hinder their practical application. Understanding the cooperative degradation mechanisms within microbial communities, leveraging multi-component technologies and synthetic biology to engineer efficient bacterial consortia for lignocellulose degradation, and optimizing fermentation processes using machine learning algorithms are crucial steps. Enhancing the catalytic efficiency and stability of cellulose-degrading enzymes through gene editing and directed evolution techniques is essential for advancing industrial applications. Furthermore, evaluating the ecological impact of large-scale microbial applications, developing cost-effective pretreatment methods, and promoting the sustainable utilization of agricultural residues are imperative for the industrialization and ecological sustainability of these technologies (Figure 4).

The technology for microbial cellulose degradation holds significant promise across various applications. Future advancements will necessitate interdisciplinary collaboration to explore microbial resources, enhance degradation techniques, facilitate industrial implementation, and offer technical assistance to achieve the high-value utilization and carbon neutrality of agricultural residues.

The integrated use of metagenomics, metatranscriptomics, and macroproteomics holds promise for identifying new cellulose-degrading microorganisms and enzymes across diverse environments, including the rumen, insect gut, and extreme ecological habitats. By leveraging CRISPR/Cas9 technology for genetic engineering, enhancements in enzyme performance, stability, and microbial consortia efficacy can be achieved, leading to enhanced lignocellulose conversion for applications like straw utilization and biomass valorization.

## Figures and Tables

**Table 3:** Isolation of cellulose-degrading bacteria from forest ecosystems.

Year	Source of Flora	Isolated Cellulose-Degrading Bacteria	Microbe Phylum, Genus	References
2018	Canadian Campus Ginkgo Grove	*Bacillus* sp. MKAL6	*Firmicutes*	[39]
2020	Qinling Mountain deadwood	*Bacillus methylotrophicus 1EJ7*	*Proteobacteria*	[40]
2019	Tropical Forest of Malaysia	*Anoxybacillus* sp. *UniMAP-KB06*	*Firmicutes*	[41]
2021	Kerman Forest, Iran	*Brevibacillus borstelensis* US5	*Firmicutes*	[42]
2021	Rice rhizosphere soil	*Massilia* sp. *NEAU-DD11T*	*Proteobacteria Massilia*	[43]
2024	Soil	*Rhodococcus wratislaviensis YZ02*, *Pseudomonas anthosomatis YZ03*	*Actinobacteria * *Proteobacteria*	[15]
2022	Mangrove soil	*Robertkochia solimangrovi*	*Bacteroidetes*	[44]

**Table 4:** Isolation of cellulose-degrading bacteria from other systems.

Year	Source of Flora	Isolated Cellulose-Degrading Bacteria	Microbe Phylum, Genus	References
2019	Banana plantation compost	*Trichoderma longibrachiatum* *Trichoderma harzianum* *Aspergillus fumigatus*	*Ascomycota*, *Trichoderma*	[47]
2021	Paper Mill Wastewater Contaminated Soil (Tanzania)	*Bacillus gemmatus* *Bacillus cereus* *Bacillus flexus*	*Firmicutes*	[48]
2024	Cow dung	*Bacillus* sp. *CD1*	*Firmicutes*	[49]

**Table 5:** Fiber-degrading enzyme systems and key applications of filamentous bacteria succinogenes, white ruminococcus, and yellow ruminococcus.

Culture	Key Enzyme Systems	Substrate Specificity	Critical Applications
*Fibrobacter succinogenes*	Cellulase system (GH family unspecified) pectinase, glucanase, arabinogalactase, xylanase	Cellulose, cellobiose, and glucose preferentially degrade cellulose-hemicellulose-pectin crosslinking network	Industrial production of succinic acid (yield 13.5 g/L) Feed additive (improve the degradation rate of neutral detergent fiber)
*Ruminococcus Albus*	GH5 family enzymes (62.3% of CAZymes) have prominent cellobiase activity	Cellobiose (38% more available than glucose) pH 6.7–5.5 optimum (less than 6.0 growth inhibition)	Complementarity with *xanthococcus* to maintain rumen microecological balance (proton power 60 mV)
*Ruminococcus flavus*	GH9 family enzymes (51.8% CAZymes). Complex enzyme system (cellulose, hemicellulose, pectin degrading enzymes)	Alkaline hydrogen peroxide treatment of wheat straw (AHPWS degradation rate 6.1 mg/d) needs phenylacetic acid/phenylacetic acid synergism	Co-culture improves fiber digestibility (e.g., *OS14 +S137 strains*) and promotes acetic acid and propionic acid production

**Table 6:** Fiber-degrading enzyme systems and key applications of white-rot fungi, brown-rot fungi, and yeast.

Culture	Key Enzyme Systems	Substrate Specificity	Critical Applications
*White-rot fungi*	Lignin-degrading enzymes (lignin peroxidase, manganese peroxidase, laccase); cellulases, hemicellulases, esterases	Preferential degradation of lignin (β-O-4′ aryl ether bond) significantly reduced lignin (*p* < 0.05) in wheat straw treated for 30 days	Feed pretreatment (increase crude protein content, metabolic energy); industrial delignification (selective degradation)
*Brown-rot fungi*	Simplified enzyme system (GH family hemicellulases, chitinases), hydroxyl radical mediated oxidative depolymerization of cellulose	Rapid depolymerization of cellulose/hemicellulose in Pinus yunnanensis preferential degradation of polysaccharides	Biomass pretreatment (to increase wood porosity) and *white-rot fungi* synergize to enhance digestibility
*Yeast*	Indirect regulation (promotion of cellulase secretion by rumen microorganisms), metabolites (e.g., short chain fatty acids)	Free sugar promotes fiber decomposition-Digestibility of crude fiber in wheat straw diet increased by 24% (*p* < 0.05)	Feed additive (increased dry matter digestibility 7.3%) improved rumen fermentation (increased TVFA, NH^3^-N)

## Data Availability

Data sharing is not applicable to this article as no datasets were generated or analyzed during the current study.

## References

[1] Zhang A., Zhang X., Liang Q., Sun M. (2024). Co-application of straw incorporation and biochar addition stimulated soil N_2_O and NH_3_ productions. PLoS ONE.

[2] Sun M., Xu X., Wang C., Bai Y., Fu C., Zhang L., Fu R., Wang Y. (2020). Environmental burdens of the comprehensive utilization of straw: Wheat straw utilization from a life-cycle perspective. J. Clean. Prod..

[3] Chen L., Hong F., Yang X.-X., Han S.-F. (2013). Biotransformation of wheat straw to bacterial cellulose and its mechanism. Bioresour. Technol..

[4] Gong X., Zou H., Qian C., Yu Y., Hao Y., Li L., Wang Q., Jiang Y., Ma J. (2020). Construction of in situ degradation bacteria of corn straw and analysis of its degradation efficiency. Ann. Microbiol..

[5] Trubetskaya A., Jensen P.A., Jensen A.D., Steibel M., Spliethoff H., Glarborg P., Larsen F.H. (2016). Comparison of high temperature chars of wheat straw and rice husk with respect to chemistry, morphology and reactivity. Biomass Bioenergy.

[6] Zhang Y., Wang H., Sun X., Wang Y., Liu Z. (2021). Separation and characterization of biomass components (cellulose, hemicellulose, and lignin) from corn stalk. BioResources.

[7] Hsu T.-C., Guo G.-L., Chen W.-H., Hwang W.-S. (2010). Effect of dilute acid pretreatment of rice straw on structural properties and enzymatic hydrolysis. Bioresour. Technol..

[8] Qiu C., Liu N., Diao X., He L., Zhou H., Zhang W. (2024). Effects of Cellulase and Xylanase on Fermentation Characteristics, Chemical Composition and Bacterial Community of the Mixed Silage of King Grass and Rice Straw. Microorganisms.

[9] Meng Z., Ma J., Sun Z., Yang C., Leng J., Zhu W., Cheng Y. (2023). Characterization of a novel bifunctional enzyme from buffalo rumen metagenome and its effect on in vitro ruminal fermentation and microbial community composition. Anim. Nutr..

[10] Fang M., Sun X., Yao F., Lu L., Ma X., Shao K., Kaimoyo E. (2024). A Combination of Transcriptome and Enzyme Activity Analysis Unveils Key Genes and Patterns of Corncob Lignocellulose Degradation by Auricularia heimuer under Cultivation Conditions. J. Fungi.

[11] Wang C., Wang Y., Ru H., He T., Sun N. (2021). Study on Microbial Community Succession and Functional Analysis during Biodegradation of Mushroom Residue. BioMed Res. Int..

[12] Mei J., Shen X., Gang L., Xu H., Wu F., Sheng L. (2020). A novel lignin degradation bacteria-Bacillus amyloliquefaciens SL-7 used to degrade straw lignin efficiently. Bioresour. Technol..

[13] Luo C., Li Y., Chen Y., Fu C., Nong X., Yang Y. (2019). Degradation of bamboo lignocellulose by bamboo snout beetle Cyrtotrachelus buqueti in vivo and vitro: Efficiency and mechanism. Biotechnol. Biofuels.

[14] Sari W.N., Safika, Darmawi, Fahrimal Y. (2017). Isolation and identification of a cellulolytic Enterobacter from rumen of Aceh cattle. Vet. World.

[15] Ma D., Chen H., Liu D., Feng C., Hua Y., Gu T., Guo X., Zhou Y., Wang H., Tong G. (2024). Soil-derived cellulose-degrading bacteria: Screening, identification, the optimization of fermentation conditions, and their whole genome sequencing. Front. Microbiol..

[16] Chen J., Zhang B., Luo L., Zhang F., Yi Y., Shan Y., Liu B., Zhou Y., Wang X., Lü X. (2021). A review on recycling techniques for bioethanol production from lignocellulosic biomass. Renew. Sustain. Energy Rev..

[17] Zhang S., Dong Z., Shi J., Yang C., Fang Y., Chen G., Chen H., Tian C. (2022). Enzymatic hydrolysis of corn stover lignin by laccase, lignin peroxidase, and manganese peroxidase. Bioresour. Technol..

[18] Falade A.O., Nwodo U.U., Iweriebor B.C., Green E., Mabinya L.V., Okoh A.I. (2017). Lignin peroxidase functionalities and prospective applications. Microbiol. Open.

[19] Atiwesh G., Parrish C.C., Banoub J., Le T.-A.T. (2022). Lignin degradation by microorganisms: A review. Biotechnol. Prog..

[20] Faruk O., Bledzki A.K., Fink H.-P., Sain M. (2012). Biocomposites reinforced with natural fibers: 2000–2010. Prog. Polym. Sci..

[21] Wohlert M., Benselfelt T., Wågberg L., Furó I., Berglund L.A., Wohlert J. (2022). Cellulose and the role of hydrogen bonds: Not in charge of everything. Cellulose.

[22] Weimer P.J. (2022). Degradation of Cellulose and Hemicellulose by Ruminal Microorganisms. Microorganisms.

[23] Wang Z.Y., Wang R.X., Zhou J.S., Cheng J.F., Li Y.H. (2020). An assessment of the genomics, comparative genomics and cellulose degradation potential of Mucilaginibacter polytrichastri strain RG4-7. Bioresour. Technol..

[24] Ríos-Ríos K.L., Dejonghe W., Vanbroekhoven K., Rakotoarivonina H., Rémond C. (2021). Enzymatic Production of Xylo-oligosaccharides from Destarched Wheat Bran and the Impact of Their Degree of Polymerization and Substituents on Their Utilization as a Carbon Source by Probiotic Bacteria. J. Agric. Food Chem..

[25] Tao J., Song S., Qu C. (2024). Recent Progress on Conversion of Lignocellulosic Biomass by MOF-Immobilized Enzyme. Polymers.

[26] Dar M.A., Pawar K.D., Pandit R.S. (2018). Prospecting the gut fluid of giant African land snail, Achatina fulica for cellulose degrading bacteria. Int. Biodeterior. Biodegrad..

[27] Oktiarni D., Hermansyah, Hasanudin, Miksusanti, Nofyan E., Kasmiarti G. (2021). Isolation and Identification Cellulolytic Bacteria from Termite Gut Obtained from Indralaya Peatland area. IOP Conf. Ser. Earth Environ. Sci..

[28] Dar M.A., Shaikh A.F., Pawar K.D., Xie R., Sun J., Kandasamy S., Pandit R.S. (2021). Evaluation of cellulose degrading bacteria isolated from the gut-system of cotton bollworm, Helicoverpa armigera and their potential values in biomass conversion. PeerJ.

[29] Nelson K., Muge E., Wamalwa B. (2021). Cellulolytic Bacillus species isolated from the gut of the desert locust Schistocerca gregaria. Sci. Afr..

[30] Silva É.B.R.d., Silva J.A.R.d., Silva W.C.d., Belo T.S., Sousa C.E.L., Santos M.R.P.d., Neves K.A.L., Rodrigues T.C.G.d.C., Camargo-Júnior R.N.C., Lourenço-Júnior J.d.B. (2024). A Review of the Rumen Microbiota and the Different Molecular Techniques Used to Identify Microorganisms Found in the Rumen Fluid of Ruminants. Animals.

[31] Morgavi D.P., Kelly W.J., Janssen P.H., Attwood G.T. (2013). Rumen microbial (meta)genomics and its application to ruminant production. Animal.

[32] Pang J., Liu Z.-Y., Hao M., Zhang Y.-F., Qi Q.-S. (2017). An isolated cellulolytic Escherichia coli from bovine rumen produces ethanol and hydrogen from corn straw. Biotechnol. Biofuels.

[33] Zhao C., Wang L., Ke S., Chen X., Kenéz Á., Xu W., Wang D., Zhang F., Li Y., Cui Z. (2022). Yak rumen microbiome elevates fiber degradation ability and alters rumen fermentation pattern to increase feed efficiency. Anim. Nutr..

[34] Guder D.G., Krishna M.S.R. (2019). Isolation and Characterization of Potential Cellulose Degrading Bacteria from Sheep Rumen. J. Pure Appl. Microbiol..

[35] Ben Ghalib K., Chadli M., Daştan S.D., Elmtili N. (2024). Isolation and molecular identification of cellulose-degrading bacteria from rumen sheep ‘’Ovis aries’’ and evaluation of their cellulase production. Sci. Afr..

[36] Yang J., Zhao J., Wang B., Yu Z. (2022). Unraveling aerobic cultivable cellulolytic microorganisms within the gastrointestinal tract of sheep (Ovis aries) and their evaluation for cellulose biodegradation. Can. J. Microbiol..

[37] Tulsani N.J., Jakhesara S.J., Hinsu A.T., Jyotsana B., Dafale N.A., Patil N.V., Purohit H.J., Joshi C.G. (2022). Genome analysis and CAZy repertoire of a novel fungus Aspergillus sydowii C6d with lignocellulolytic ability isolated from camel rumen. Electron. J. Biotechnol..

[38] Pramartaa I.Q., Wiryawan K.G., Suharti S. (2024). Microbial Protein Synthesis by Cellulolytic Bacterial Isolates from Feces of Indonesian Endemic Herbivores. Indones. J. Appl. Res. (IJAR).

[39] Mokale Kognou A.L., Chio C., Khatiwada J.R., Shrestha S., Chen X., Han S., Li H., Jiang Z.-H., Xu C.C., Qin W. (2022). Characterization of Cellulose-Degrading Bacteria Isolated from Soil and the Optimization of Their Culture Conditions for Cellulase Production. Appl. Biochem. Biotechnol..

[40] Ma L., Lu Y., Yan H., Wang X., Yi Y., Shan Y., Liu B., Zhou Y., Lü X. (2020). Screening of cellulolytic bacteria from rotten wood of Qinling (China) for biomass degradation and cloning of cellulases from Bacillus methylotrophicus. BMC Biotechnol..

[41] Naresh S., Kunasundari B., Gunny A.A.N., Teoh Y.P., Shuit S.H., Ng Q.H., Hoo P.Y. (2019). Isolation and Partial Characterisation of Thermophilic Cellulolytic Bacteria from North Malaysian Tropical Mangrove Soil. Trop. Life Sci. Res..

[42] Khosravi F., Khaleghi M., Naghavi H. (2021). Screening and identification of cellulose-degrading bacteria from soil and leaves at Kerman province, Iran. Arch. Microbiol..

[43] Du C., Li C., Cao P., Li T., Du D., Wang X., Zhao J., Xiang W. (2021). Massilia cellulosiltytica sp. nov., a novel cellulose-degrading bacterium isolated from rhizosphere soil of rice (*Oryza sativa* L.) and its whole genome analysis. Antonie Van Leeuwenhoek.

[44] Lam M.Q., Oates N.C., Leadbeater D.R., Goh K.M., Yahya A., Md Salleh M., Ibrahim Z., Bruce N.C., Chong C.S. (2022). Genomic Analysis to Elucidate the Lignocellulose Degrading Capability of a New Halophile Robertkochia solimangrovi. Genes.

[45] Hao X., Jianzheng L., Jun X., Yiyang F., Furao W., Meng J. (2025). Isolation and characterization of a low-temperature and cellulose-degrading fungus *Tausonia pullulans* LC-6. Environ. Technol..

[46] Li K., Fu S., Zhan H., Zhan Y., Lucia L.A. (2010). Analysis of the chemical composition and morphological structure of banana pseudo-stem. BioResources.

[47] Legodi L.M., La Grange D., van Rensburg E.L.J., Ncube I. (2019). Isolation of Cellulose Degrading Fungi from Decaying Banana Pseudostem and Strelitzia alba. Enzym. Res..

[48] Sinza E., Mwakilili A., Mpinda C., Lyantagaye S. (2021). Cellulase-producing bacteria isolated from Mufindi Paper Mill industrial effluent, Iringa Tanzania. Tanzan. J. Sci..

[49] Demissie M.S., Legesse N.H., Tesema A.A. (2024). Isolation and characterization of cellulase producing bacteria from forest, cow dung, Dashen brewery and agro-industrial waste. PLoS ONE.

[50] Hussain A.A., Abdel-Salam M.S., Abo-Ghalia H.H., Hegazy W.K., Hafez S.S. (2017). Optimization and molecular identification of novel cellulose degrading bacteria isolated from Egyptian environment. J. Genet. Eng. Biotechnol..

[51] Khatri D., Chhetri S.B.B. (2020). Reducing Sugar, Total Phenolic Content, and Antioxidant Potential of Nepalese Plants. BioMed Res. Int..

[52] Lanjekar V.B., Hivarkar S.S., Vasudevan G., Joshi A., Dhakephalkar P.K., Dagar S.S. (2022). Actinomyces ruminis sp. nov., an obligately anaerobic bacterium isolated from the rumen of cattle. Arch. Microbiol..

[53] Stewart R.D., Auffret M.D., Warr A., Walker A.W., Roehe R., Watson M. (2019). Compendium of 4941 rumen metagenome-assembled genomes for rumen microbiome biology and enzyme discovery. Nat. Biotechnol..

[54] Lagier J.C., Armougom F., Million M., Hugon P., Pagnier I., Robert C., Bittar F., Fournous G., Gimenez G., Maraninchi M. (2012). Microbial culturomics: Paradigm shift in the human gut microbiome study. Clin. Microbiol. Infect..

[55] Kaminski T.S., Scheler O., Garstecki P. (2016). Droplet microfluidics for microbiology: Techniques, applications and challenges. Lab A Chip.

[56] Villa M.M., Bloom R.J., Silverman J.D., Durand H.K., Jiang S., Wu A., Dallow E.P., Huang S., You L., David L.A. (2020). Interindividual Variation in Dietary Carbohydrate Metabolism by Gut Bacteria Revealed with Droplet Microfluidic Culture. mSystems.

[57] Zehavi T., Probst M., Mizrahi I. (2018). Insights Into Culturomics of the Rumen Microbiome. Front. Microbiol..

[58] Zhang Z., Li F., Ma X., Li F., Wang Z. (2022). Effects of Barley Starch Level in Diet on Fermentation and Microflora in Rumen of Hu Sheep. Animals.

[59] Lee P., Lee S., Hong S., Chang H. (2002). Isolation and characterization of a new succinic acid-producing bacterium, Mannheimia succiniciproducens MBEL55E, from bovine rumen. Appl. Microbiol. Biotechnol..

[60] Yeoman Carl J., Fields Christopher J., Lepercq P., Ruiz P., Forano E., White Bryan A., Mosoni P. (2021). In Vivo Competitions between Fibrobacter succinogenes, Ruminococcus flavefaciens, and Ruminoccus albus in a Gnotobiotic Sheep Model Revealed by Multi-Omic Analyses. mBio.

[61] Dassa B., Borovok I., Ruimy-Israeli V., Lamed R., Flint H.J., Duncan S.H., Henrissat B., Coutinho P., Morrison M., Mosoni P. (2014). Rumen Cellulosomics: Divergent Fiber-Degrading Strategies Revealed by Comparative Genome-Wide Analysis of Six Ruminococcal Strains. PLoS ONE.

[62] Boonsaen P., Poonko S., Kanjanapruetipong J., Phiriyangkul P., Sawanon S. (2019). Isolation and partial characterization of Ruminococcus flavefaciens from the rumen of swamp buffalo. Buffalo Bull..

[63] Odenyo A.A., Mackie R.I., Fahey G.C., White B.A. (1991). Degradation of wheat straw and alkaline hydrogen peroxide-treated wheat straw by Ruminococcus albus 8 and Ruminococcus flavefaciens FD-1. J. Anim. Sci..

[64] van Erven G., Nayan N., Sonnenberg A.S.M., Hendriks W.H., Cone J.W., Kabel M.A. (2018). Mechanistic insight in the selective delignification of wheat straw by three white-rot fungal species through quantitative 13C-IS py-GC–MS and whole cell wall HSQC NMR. Biotechnol. Biofuels.

[65] Arora D.S., Chander M., Gill P.K. (2002). Involvement of lignin peroxidase, manganese peroxidase and laccase in degradation and selective ligninolysis of wheat straw. Int. Biodeterior. Biodegrad..

[66] Tabka M.G., Herpoël-Gimbert I., Monod F., Asther M., Sigoillot J.C. (2006). Enzymatic saccharification of wheat straw for bioethanol production by a combined cellulase xylanase and feruloyl esterase treatment. Enzym. Microb. Technol..

[67] Shrivastava B., Thakur S., Khasa Y.P., Gupte A., Puniya A.K., Kuhad R.C. (2011). White-rot fungal conversion of wheat straw to energy rich cattle feed. Biodegradation.

[68] van Erven G., Wang J., Sun P., de Waard P., van der Putten J., Frissen G.E., Gosselink R.J.A., Zinovyev G., Potthast A., van Berkel W.J.H. (2019). Structural Motifs of Wheat Straw Lignin Differ in Susceptibility to Degradation by the White-Rot Fungus Ceriporiopsis subvermispora. ACS Sustain. Chem. Eng..

[69] Arantes V., Jellison J., Goodell B. (2012). Peculiarities of brown-rot fungi and biochemical Fenton reaction with regard to their potential as a model for bioprocessing biomass. Appl. Microbiol. Biotechnol..

[70] Eastwood D.C., Floudas D., Binder M., Majcherczyk A., Schneider P., Aerts A., Asiegbu F.O., Baker S.E., Barry K., Bendiksby M. (2011). The Plant Cell Wall–Decomposing Machinery Underlies the Functional Diversity of Forest Fungi. Science.

[71] Gaskell J., Blanchette R.A., Stewart P.E., BonDurant S.S., Adams M., Sabat G., Kersten P., Cullen D. (2016). Transcriptome and Secretome Analyses of the Wood Decay Fungus Wolfiporia cocos Support Alternative Mechanisms of Lignocellulose Conversion. Appl. Environ. Microbiol..

[72] Qi J., Zhang X., Zhou Y., Zhang C., Wen J., Deng S., Luo B., Fan M., Xia Y. (2023). Selectively enzymatic conversion of wood constituents with white and brown rot fungi. Ind. Crops Prod..

[73] Cömert M., Şayan Y., Özelçam H., Baykal G.Y. (2015). Effects of Saccharomyces cerevisiae Supplementation and Anhydrous Ammonia Treatment of Wheat Straw on In-situ Degradability and, Rumen Fermentation and Growth Performance of Yearling Lambs. Asian-Australas J. Anim. Sci..

[74] Ben Saïd S., Jabri J., Amiri S., Aroua M., Najjar A., Khaldi S., Maalaoui Z., Kammoun M., Mahouachi M. (2022). Effect of Saccharomyces cerevisiae Supplementation on Reproductive Performance and Ruminal Digestibility of Queue Fine de l’Ouest Adult Rams Fed a Wheat Straw-Based Diet. Agriculture.

[75] Ding G., Chang Y., Zhao L., Zhou Z., Ren L., Meng Q. (2014). Effect of Saccharomyces cerevisiae on alfalfa nutrient degradation characteristics and rumen microbial populations of steers fed diets with different concentrate-to-forage ratios. J. Anim. Sci. Biotechnol..

[76] Bai D.-P., Lin X.-Y., Hu Y.-Q., Chen Z.-Z., Chen L., Huang Y.-F., Huang X.-H., Li J. (2021). Metagenomics approach to identify lignocellulose-degrading enzymes in the gut microbiota of the Chinese bamboo rat cecum. Electron. J. Biotechnol..

[77] Chai S., Zhang X., Jia Z., Xu X., Zhang Y., Wang S., Feng Z. (2020). Identification and characterization of a novel bifunctional cellulase/hemicellulase from a soil metagenomic library. Appl. Microbiol. Biotechnol..

[78] Wang L., Hatem A., Catalyurek U.V., Morrison M., Yu Z. (2013). Metagenomic Insights into the Carbohydrate-Active Enzymes Carried by the Microorganisms Adhering to Solid Digesta in the Rumen of Cows. PLoS ONE.

[79] He B., Jin S., Cao J., Mi L., Wang J. (2019). Metatranscriptomics of the Hu sheep rumen microbiome reveals novel cellulases. Biotechnol. Biofuels.

[80] Yu W., Wu Y., Li D. (2025). Oxidative cleavage of cellulose by fungi in the termite gut. Int. J. Biol. Macromol..

[81] Xie M., An F., Wu J., Liu Y., Shi H., Wu R. (2019). Meta-omics reveal microbial assortments and key enzymes in bean sauce mash, a traditional fermented soybean product. J. Sci. Food Agric..

[82] Lee S.H., Whon T.W., Roh S.W., Jeon C.O. (2020). Unraveling microbial fermentation features in kimchi: From classical to meta-omics approaches. Appl. Microbiol. Biotechnol..

[83] Toyoda A., Iio W., Mitsumori M., Minato H. (2009). Isolation and Identification of Cellulose-Binding Proteins from Sheep Rumen Contents. Appl. Environ. Microbiol..

[84] Liu Q., Gao R., Li J., Lin L., Zhao J., Sun W., Tian C. (2017). Development of a genome-editing CRISPR/Cas9 system in thermophilic fungal Myceliophthora species and its application to hyper-cellulase production strain engineering. Biotechnol. Biofuels.

[85] Liu G., Gong H., Tang H., Meng Z., Wang Z., Cui W., Zhang K., Chen Y., Yang Y. (2025). Enhanced lignocellulose degradation in Bacillus subtilis RLI2019 through CRISPR/Cas9-mediated chromosomal integration of ternary cellulase genes. Int. J. Biol. Macromol..

[86] Zhou C., Zhou H., Li D., Zhang H., Wang H., Lu F. (2020). Optimized expression and enhanced production of alkaline protease by genetically modified Bacillus licheniformis 2709. Microb. Cell Factories.

